# An online mapping database of molecular markers of drug resistance in *Plasmodium falciparum*: the ACT Partner Drug Molecular Surveyor

**DOI:** 10.1186/s12936-019-2645-x

**Published:** 2019-01-18

**Authors:** Sabina Dahlström Otienoburu, Ignacio Suay, Steven Garcia, Nigel V. Thomas, Suttipat Srisutham, Anders Björkman, Georgina S. Humphreys

**Affiliations:** 1 WorldWide Antimalarial Resistance Network (WWARN), Oxford, UK; 2 Department of Computer Science and Engineering, Johnson C. Smith University, Charlotte, NC USA; 3 Department of Molecular Tropical Medicine and Genetics, Faculty of Tropical Medicine, Mahidol University, Bangkok, 10400 Thailand; 4 Department of Microbiology, Tumour and Cell Biology, Karolinska Institutet, Stockholm, Sweden

**Keywords:** Malaria, Drug resistance, Molecular markers, *pfmdr1*, *pfcrt*, Geovisualization

## Abstract

**Background:**

Prior to this project, only a handful of online visualizations existed for exploring the published literature on molecular markers of antimalarial drug resistance, and none specifically for the markers associated with *Plasmodium falciparum* resistance to the partner drugs in artemisinin-based combination therapy (ACT). Molecular information is collected in studies with different designs, using a variety of molecular methodologies and data analysis strategies, making it difficult to compare across studies. The purpose of this project was to develop a free online tool, which visualizes the widely published data on molecular markers of antimalarial drug resistance, starting with the two genes *pfcrt* and *pfmdr*-*1*, associated with resistance to the three most common partner drugs; amodiaquine, lumefantrine and mefloquine.

**Methods:**

A literature review was conducted, and a standardized method was used to extract data from publications, and critical decisions on visualization were made. A global geospatial database was developed of specific *pfmdr1* and *pfcrt* single nucleotide polymorphisms and *pfmdr1* copy number variation. An informatics framework was developed that allowed flexibility in development of the tool over time and efficient adaptation to different source data.

**Results:**

The database discussed in this paper has *pfmdr1* and *pfcrt* marker prevalence information, from 579 geographic sites in 76 different countries, including results from over 86,000 samples from 456 articles published January 2001–May 2017. The ACT Partner Drugs Molecular Surveyor was launched by the WorldWide Antimalarial Resistance Network (WWARN) in March 2015 and it has attracted over 3000 unique visitors since then. Presented here is a demonstration of how the Surveyor database can be explored to monitor local, temporal changes in the prevalence of molecular markers. Here publications up to May 2017 were included, however the online ACT partner drug Molecular Surveyor is continuously updated with new data and relevant markers.

**Conclusions:**

The WWARN ACT Partner Drugs Molecular Surveyor summarizes data on resistance markers in the *pfmdr1* and *pfcrt* genes. The database is fully accessible, providing users with a rich resource to explore and analyze, and thus utilize a centralized, standardized database for different purposes. This open-source software framework can be adapted to other data, as demonstrated by the subsequent launch of the Artemisinin Molecular Surveyor and the Vivax Surveyor.

**Electronic supplementary material:**

The online version of this article (10.1186/s12936-019-2645-x) contains supplementary material, which is available to authorized users.

## Background

Malaria remains a major cause of morbidity and mortality with a concerning trend of increase in cases reported in the 2017 World Malaria Report (216 million cases of malaria in 2016, up 5 million from 2015) [1]. Africa still bears most of the burden with 90% of all malaria cases and deaths occurring in this region [1].

The continuing devastating impact of this disease is partly due to the emergence and spread of resistance to anti-malarials. Mutations in *Plasmodium falciparum* genes have long been established as markers of anti-malarial resistance [2–5], and the level of clinical treatment failure has been associated with the presence of these resistance mutations [6, 7]. Identification of both copy number variation (CNV) and single nucleotide polymorphisms (SNPs) in the *P. falciparum* genes allows monitoring of the emergence and spread of declining drug susceptibility in parasite populations [8].

With respect to the partner drugs used in currently recommended artemisinin-based combination therapy (ACT), resistance to amodiaquine has been associated with SNPs in both the *P. falciparum* chloroquine-resistance transporter (*pfcrt*) MAL7P1.27 and *P. falciparum* multidrug resistance gene 1 (*pfmdr1*) MAL5P1.230 [6, 7, 9] (Table 1). Increased risk of treatment failure after lumefantrine treatment [7] and decreased susceptibility to lumefantrine in vitro are associated with several specific alleles of *pfmdr1* [10–12]. Resistance to mefloquine has been associated with amplification of the *pfmdr1* gene [13].

**Table 1:** SNPs and copy number variations associated with resistance to ACT partner drugs

	Lumefantrine	Amodiaquine	Chloroquine	Mefloquine
*pfmdr1* N86Y	N86	86Y	86Y	
*pfmdr1* Y184F	184F	Y184		
*pfmdr1* D1246Y	D1246	1246Y		
*pfmdr1* copy no.				↑ Increased CN
*pfcrt* K76T	K76	76T	76T	
*pfcrt* 72–76	CxxxK	SVMNT	CVIET	

Molecular surveillance can be used to estimate the impact of parasite resistance on preventative measures such as intermittent preventive therapy in pregnancy (IPTp) and seasonal malaria chemoprevention (SMC), as well as to assess the appropriateness of current recommended drug treatment policies [14–16]. One example of drug policy change having an impact on the prevalence of particular markers of resistance was observed in Tanzania, within 4 years from the introduction of artemether–lumefantrine (AL) as the first-line treatment in 2006, the prevalence of the *pfmdr1* N86 and 184F alleles increased dramatically from 10% to 46% and < 10% to 40%, respectively [17]. Such evolution has direct consequences for patient treatment since the prevalence of a particular genotype in the parasite population is one factor considered in predicting an overall probability of treatment success [8].

It is presently still uncommon that treatment policies have been changed based on molecular studies alone, but two examples, from Mali and Tanzania, have demonstrated the use of local marker prevalences in evaluating drug policies [8, 18]. On a global level, mutations in *P. falciparum dihydrofolate reductase (pfdhfr)*, PF3D7_0417200, and *P. falciparum dihydropteroate synthase (pfdhps)*, PF3D7_0810800, markers of sulfadoxine-pyrimethamine (SP) resistance, played a role in the World Health Organization (WHO) decision to change IPTp policy recommendations from a 2-dose regimen of SP to SP administration at each scheduled antenatal care visit [19, 20].

The Millennium Development Goals Report 2015 highlights the importance of data collection and its use in evidence-based policymaking, recommending tailored local strategies and “opening up data… to provide free visualization and analysis tools” [21]. However, very few interactive maps that provide a summary of the anti-malarial drug resistance picture exist online, and none that allow the user to explore temporal trends. The London School of Hygiene and Tropical Medicine Africa map displays prevalence of mutations in the *pfdhfr* and *pfdhps* genes associated with SP resistance (http://www.drugresistancemaps.org). Another example is the Center for Global Development drug resistance maps which display the number of cases of CQ or SP resistance at the country level (http://www.cgdev.org/page/looking-drug-resistance). The more recently launched WHO Malaria Threats Map [22] presents information on resistance markers in the *Pfkelch* 13, *pfcrt, pfmdr-1,* and *pfplasmepsin 2-3 genes* (http://www.who.int/malaria/maps/threats). There are other examples of informative maps on malaria but these do not present drug resistance data (CDC interactive map (http://www.cdc.gov/malaria/map/index.html), and the Malaria Atlas Project (http://www.map.ox.ac.uk).

There is evidence of heterogeneity in marker prevalences between populations sampled from neighbouring regions within a country [23] which challenges the degree of spatial generalizability one can draw from a particular sample site. This highlights the need to make the most of all the data that do exist and supports the development of a single comprehensive source of all the evidence collected to summarize the data and to highlight gaps. This allows targeting of control programme resources to the areas of highest concern.

The WorldWide Antimalarial Resistance Network (WWARN) recognizes that data often need transforming into formats that are useful for a wide audience [24]. WWARN created an interactive map for point mutations in the *pfdhfr* and *pfdhps* genes (http://www.wwarn.org/dhfr-dhps-surveyor/). The next step was to summarize the published evidence on mutations in the *pfcrt* and *pfmdr1* genes which have been associated with resistance to chloroquine (CQ) and the main ACT partner drugs, namely amodiaquine (AQ), lumefantrine (LUM), and mefloquine (MQ).

Collating the current evidence in a standardized approach is a key first step to facilitate the consideration of molecular marker prevalence evidence in informed treatment policies. The aim of this current effort was to conduct a periodic review of the published literature and create a useful, interactive visualization tool to summarize the information both spatially and temporally. WWARN’s intention is to present data without extrapolation or interpretation, using a clear, transparent methodology.

## Methods

### Data search and inclusion and exclusion criteria for publications

Studies have been identified every 6 months since 2015 through a PubMed literature review using the search terms ‘malaria AND (*pfcrt* OR *pfmdr1* OR “molecular marker” OR “molecular markers”)’. The ACT partner drug Surveyor is regularly updated with newly published or unpublished data (minimum every 6 months). Articles published from 2001 onwards are included. Abstracts and text are scanned to determine which publications are relevant using the following inclusion criteria; reporting of at least one *pfcrt* or *pfmdr1* genotype or haplotype from field isolates, i.e. not cultured strains (see Table 2 for a complete list of markers) and clear information on sample size, and location information (at least on country level). If malaria patient samples are assessed, only results from pre-treatment samples are considered. Details of the inclusion and exclusion criteria can be found in the Additional file 1. In this publication, data from articles published between January 2001 and May 2017 are presented. A full list of the publications captured in the search can be found in the Additional file 2. Future literature searches will also include prevalence data on plasmepsin2 CNV, related to piperaquine resistance [25, 26]. The ACT Partner Drug Molecular Surveyor is continuously updated with data from publications.

**Table 2:** SNPs, haplotypes displayed by codon number and CNV displayed in the ACT Partner Drug Molecular Surveyor

*pfmdr1*
N86Y
Y184F
D1246Y
N86Y/Y184F/D1246Y
N86Y/Y184F/S1034C/N1042D/D1246Y
copy number
*pfcrt*
K76T
S72C/M74I/N75E/K76T

### Data extraction and entry in the database

Data extracted from the publications includes marker genotype and haplotype prevalence (number of samples tested, number of samples with the pure and/or mixed genotype identified) and gene copy number as well as PubMedID and publication information, study year and geographic position (country, site, latitude and longitude). Data on multiple loci in *pfmdr1* and *pfcrt* are extracted to determine the prevalence of genotypes and haplotypes (Table 2). If latitude/longitude were not reported in the article, geocoding was performed using online tools (http://www.gpsvisualizer.com/geocoder/ or http://www.google.com/maps).

An online web application has been developed to increase consistency in data entry and to support entry concurrently by multiple staff based anywhere in the world. Each user connects to the application through secure controlled access and each interaction with the application is audited. The main WWARN SQL database is a fault-tolerant database, which replicates data in different data centres to achieve high availability and durability. Daily back-ups are also applied in order to minimize data loss.

The following rules guide the process of data extraction and entry:

- Data are extracted and entered per site per year, if this information is available.
- Sample size must be specified per site and marker.
- Articles are scanned particularly to identify reports of polyclonal infections. If polyclonal infections are present in the data set, they can be entered separately, or included in either the mutant and wild type categories or both, depending on the information given in the publication.
- If no sample collection date is reported, the year of sample collection is estimated to 3 years prior to the publication year.
- If the prevalence of only one allele (e.g. *pfcrt* 76T) is presented in the publication, the prevalence of the other allele (*pfcrt* K76) is calculated from the presented prevalence and extracted, if the mixed infections (*pfcrt* 76 K/T) were clearly accounted for.
- If the marker prevalence is only presented in a graph in the article, the prevalence for each genotype is estimated based on the graph.
- If only the prevalence of haplotypes (e.g. *pfcrt* 72–76) is presented in the publication, the prevalence of the single locus (*pfcrt* 76) is calculated from the haplotype prevalence, if all identified haplotypes were accounted for in the publication.

### The online visualization tool

The Surveyor tool was built using the Google Web Toolkit, Bootstrap, a responsive front-end library and the WWARN maps surveyor open source framework (https://github.com/WorldwideAntimalarialResistanceNetwork/WWARN-Maps-Surveyor). This framework is a mapping platform for visualizing data to support research synthesis and reporting in global health settings and health research. The tool displays the prevalence of a molecular marker including mixed infections for both wild-type and mutant alleles, by location. Data can be filtered to view results on a particular ACT-partner drug, molecular marker, country, sample size, and sample collection year. Pin icons display the location of samples collected as reported in the publications and are coloured according to the prevalence of the marker being viewed. Pop-up boxes provide specific details about samples from a particular site. These boxes include a direct citation link to PubMed. If samples from more than 1 year have been reported from that site, a drop down menu allows each year to be viewed separately. The prevalence of molecular markers in two geographic sites or the same geographic site from different years can be compared with a tool below the map by selecting country, site and year from the drop-down menus. The full dataset can be downloaded as a single comma-separated value (csv) file which can be input for analysis in excel or other statistical software. The dataset that the user downloads can be customized to reflect the filter choices applied by the user.

Rules for data visualization:

- For a study with multiple geographic sites, where the data cannot be separated by site, the data are displayed as all geographic sites together with the pin pointing at one of the sites.
- If only the country of origin, but not the study site is specified, the data are displayed with a pin pointing at the capital of the country.
- If a study collected samples over several years, the study is displayed with one pin coloured according to the data from the most recent year.
- Inclusion or exclusion of mixed infections in the prevalence of a single genotype or haplotype is specified in the pop-up box.

Although there are 29 different loci (17 in *pfcrt*, and 12 in *pfmdr1*) captured in the database, the majority of the data concentrate on a few key loci; *pfmdr1* codons 86, 184, 1246, and CNV; and *pfcrt* codons 72–76. To simplify visualization of the data on the Surveyor, only these key positions are listed in the marker list as they represent both the majority of the data, and the loci with the clearest evidence for a role in drug resistance. In addition to single nucleotide polymorphisms, haplotypes are also presented. The prevalence of particular single SNPs from data on haplotypes has been included in the single SNP prevalence. For example, if the user chooses ‘*pfcrt* 72–76 CXXXT’, where X represents any amino acid, then all publications that reported any haplotype will be displayed. In addition the prevalence of *pfcrt* 76T will be calculated based on all haplotypes presented in the article and displayed when the user chooses *pfcrt* 76T.

### Analysis of the surveyor database

The Surveyor data set, openly available to download, can be used to provide visualization of spatial and temporal changes in marker prevalence over time. In the example presented in the paper, the data set was analysed to show the number of publications by year and region (Figs. 2, 3) as well two examples of temporal and spatial changes of marker prevalence. Firstly, temporal changes in the prevalence of *pfcrt* K76 and *pfmdr1* N86 in a specific site, Tororo, Uganda, were addressed. The prevalence by year of the SNPs was calculated by dividing the sum of samples positive for a genotype (including mixed infections) for all studies in Tororo by the sum of tested samples (Fig. 5). Secondly, spatial and temporal changes of *pfmdr1* N86 prevalence in six selected sub-Saharan countries were visualized (Fig. 6). The SNP prevalence from each of 42 studies was plotted by year and geographical site and colored by country. A line marked the mean prevalence of all study sites in a country by year. The mean was calculated by dividing the sum of samples positive for *pfmdr1* N86 (including 86 N + Y) for all studies divided by the sum of tested samples by year, and presented based on the current first-line ACT in the country, according to WHO [2]. Selected sub-Saharan countries with data from > 50 samples in each site conducted between 2000 and 2015 were included. If studies were conducted over a range of years, the mean year was used. Countries were selected for analysis based on number of data points and spread over time. Data from Zanzibar were presented separately from Tanzania mainland since different first-line treatments were adopted in these areas. Fisher’s exact two-tailed test was used to compare the prevalence of the binned year groups 2003–2006 and 2009–2012. A full reference list of the original source publications used to create Fig. 6 can be found in Additional file 3. Graphs were created in Excel and Tableau version 10.5.

## Results

### The database

**Fig. 1:**
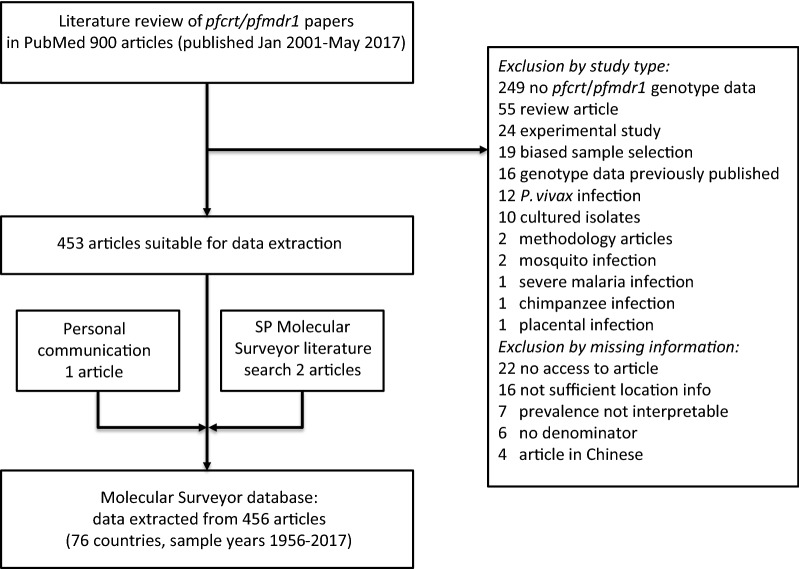
Flow diagram outlining study selection

The database presented here included data from articles published between January 2001 and May 2017. The literature search identified 903 papers of which 450 (50%) were excluded (Fig. 1). Excluded studies fell into two groups; inappropriate study type and missing information. Within the study type category, most studies did not collect data of baseline prevalence in *pfcrt* and *pfmdr1* markers (n = 249, 55%). This was expected as wide search terms were deliberately chosen to capture the majority of publications containing relevant data. Within the missing information category, 16 (4%) publications had unclear sample location details and six publications had an unspecified total number of samples analysed. Among the included studies 46 (10%) did not contain information on the year of sample collection. The sample collection year was then set to 3 years prior to publication. A total of 453 articles were included. In addition, one article was identified by personal communication and two articles in the PubMed literature search for the “SP Molecular Surveyor” since they included data on the ACT partner drug markers. Data have thus been extracted from 456 publications, which contained results from over 86,000 samples. A full list of the publications captured in the search can be found in the Additional file 2.

**Fig. 2:**
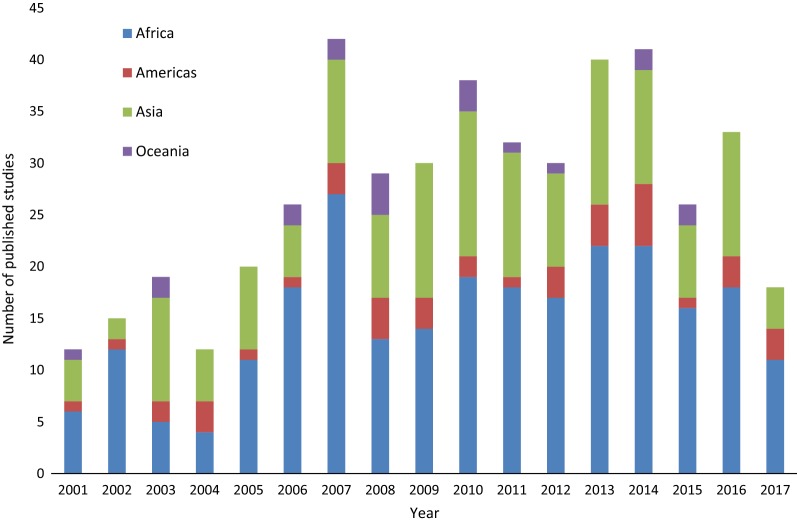
Number of studies published each year, coloured by region where samples were collected

A range of 12 to 42 publications were included from each year between 2001 and May 2017 (Fig. 2). Samples had been collected between 1956 and 2017 and the median time between final sample collection and publication was 3 years, with a range of 0–50 years.

**Fig. 3:**
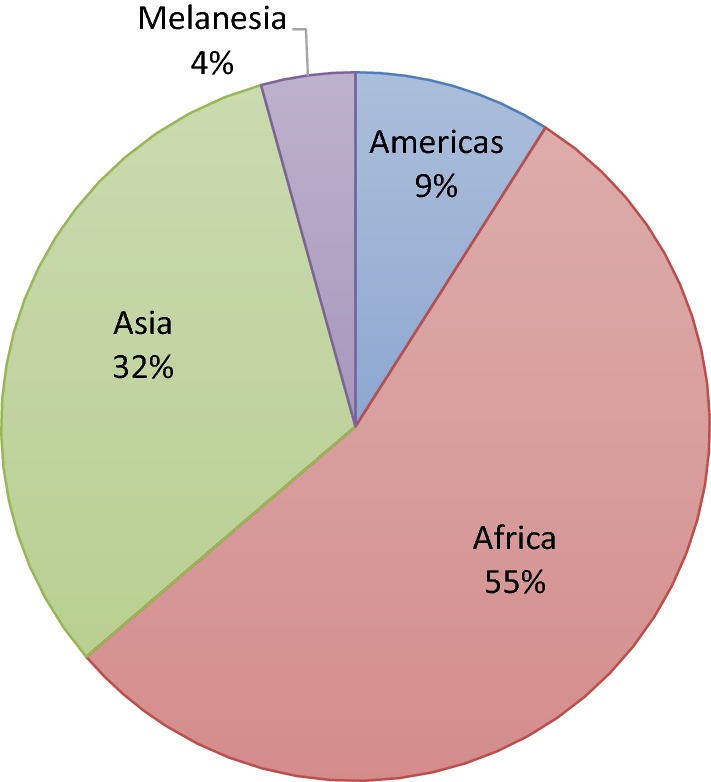
Pie chart displaying where samples were collected, pooling years 1956–2017

Studies were conducted in 76 different countries, covering a total of 579 geographic sites. Just over half of the studies reported data from at least one site in Africa (55% of publications) and 32% had at least one site in Asia (see Fig. 3). The vast majority of the publications (389, 86%) reported results for *pfcrt* with 141 reporting haplotypes for *pfcrt72*–*76*. A total of 330 papers (73%) reported any alleles in *pfmdr1* and 87% of those (287) reported the *pfmdr1 86* allele. Only 47 studies reported haplotypes for at least the three most commonly genotyped *pfmdr1* loci (*86/184/1246). Pfmdr1* copy number was reported in 84 (19%) publications.

**Fig. 4:**
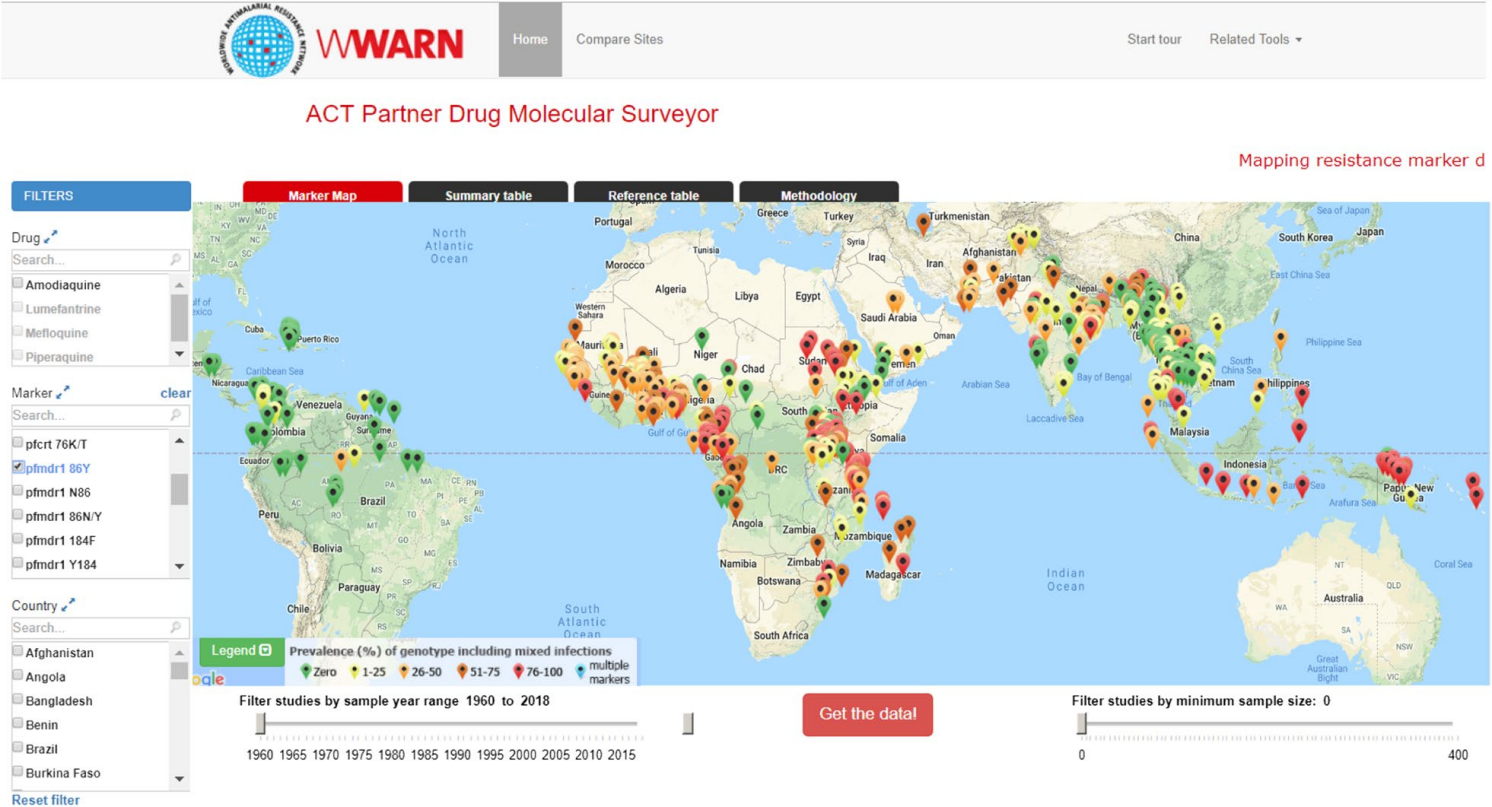
Screen shot of Surveyor, displaying data on the *pfmdr1* 86Y mutation allele

The extracted data from all 456 publications are now presented as an online tool [27] entitled the *WWARN ACT Partner Drugs Molecular Surveyor* (Fig. 4). This interactive map allows users to explore the data visually using filters to select data by drug, marker, country, year of sampling, and sample size. Direct comparison between geographic sites, and at a single site between different years, can be undertaken in a special section below the main map.

### Temporal and spatial trends of molecular markers

**Fig. 5:**
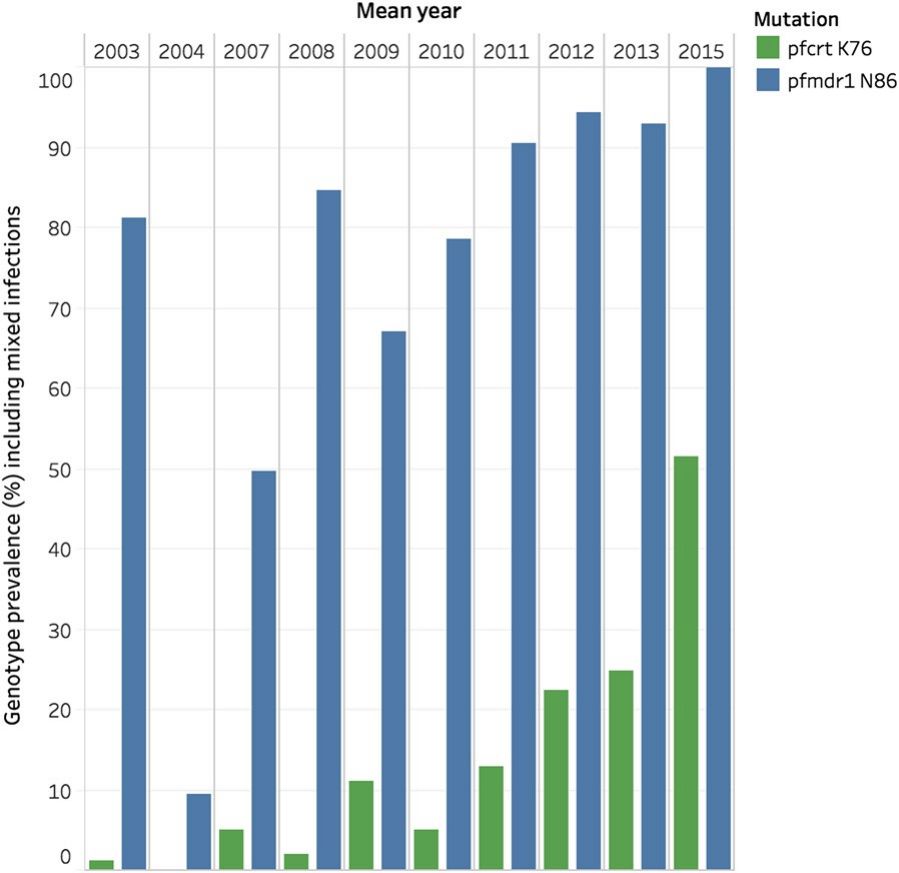
Temporal changes in prevalence of* pfmdr1* N86 and *pfcrt* K76 Tororo, Uganda

**Fig. 6:**
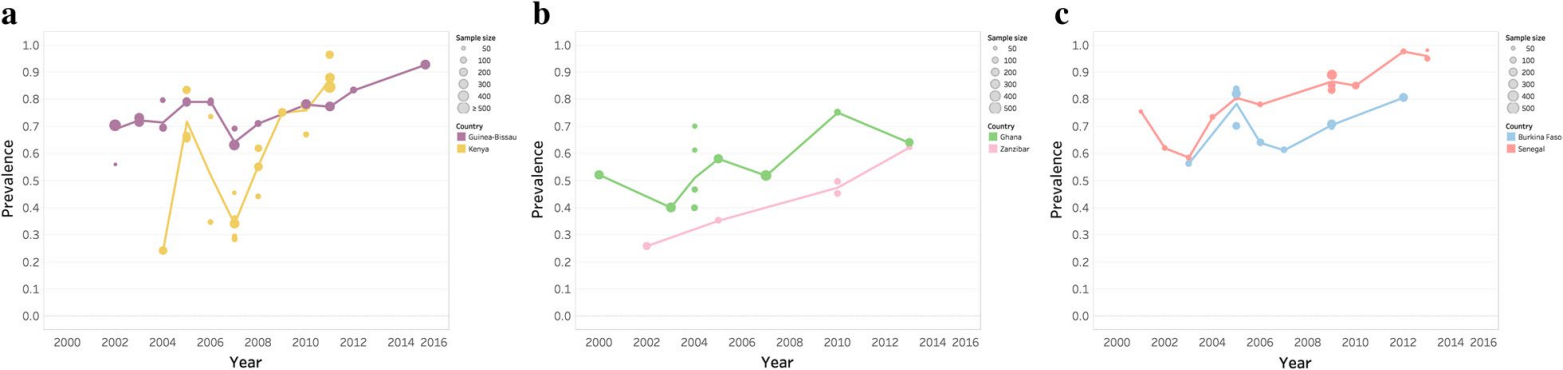
Prevalence of *pfmdr1* N86 in sub-Saharan countries by study and year. Each point depicts one study, sized according to the number of tested samples. The line represents the annual mean of all studies in a country. Mixed infections 86 N + Y were included together with N86 in the analysis. The countries either have** a** AL as first-line treatment,** b** ASAQ as first-line treatment or** c** multiple first line treatments. This data was extracted from 42 source publications, details of which can be found in Additional file 3

The Molecular Surveyor can be used to see changes over time in specific geographic sites or to visualize spatial and temporal trends of molecular marker prevalence on a local, national or global level. To illustrate the kinds of secondary studies that can be easily undertaken with the data freely available in the surveyor database, a detailed description of the data from Tororo, Uganda was prepared. Tororo was chosen, as it was the site in the database with the richest data set over time. Changes in the prevalence of markers *pfmdr1* N86 and *pfcrt* K76 (both associated with resistance/tolerance to lumefantrine) were visualized, using samples spanning 2002 to 2015 (Fig. 5). These data demonstrate the power of bringing multiple datasets together as information is pooled from eight different publications including results from 3405 samples [28–35]. To investigate national trends, temporal *pfmdr1* N86 prevalences were visualized in six sub-Saharan countries between 2000 and 2015 (Fig. 6). ACT was adopted as first-line therapy for uncomplicated malaria between 2003 and 2006 in sub-Saharan countries [36], which are presented by first-line treatment. The prevalence between binned years 2003–2006 and 2009–2012 were compared using Fisher’s exact test. The results suggest that *pfmdr1* N86 prevalence increased significantly in all countries studied.

## Discussion

The aim is for the ACT Partner Drug Molecular Surveyor to be a dynamic tool updated with newly published data and new markers that will engage researchers. Additional information may need to be requested from investigators in order for the data set to be included in the Surveyor. The goal is that the surveyor can be a tool that will be useful to a broad range of users and data providers. This group could then work collaboratively to produce an increasingly integrated/coordinated surveyor system. Further development work may involve providing an even more detailed provision of information for local adjustment of optimal drug policies within a country.

Due to the large heterogeneity in reporting of molecular marker results, WWARN developed clear rules for extraction, including how to handle missing information, to complete the data extraction process. This standardized approach means that the resulting dataset is consistent and allow comparison between studies, although it also requires that some published data cannot be included due to the lack of reported details. Reporting of a minimum set of variables including sample size, study site, year of collection and how mixed infections are handled, would enable inclusion of more data in the future. Investigators are encouraged to include these data in each new manuscript, even if they have been specified in a previous publication.

Analysis of the Surveyor database can provide valuable insight in marker prevalence dynamics. As an example, and interestingly, molecular marker *pfmdr1* N86 increased in six countries in sub-Saharan Africa between 2000 and 2016, independent of the adopted first-line treatment (Fig. 6). Overall, the data suggest there are factors other than/in addition to first-line treatment that govern prevalences and distributions of alleles. National, local and temporal policy variations of ACT deployment/use could occur. In the studied countries ACT use differed as much as from below 10% to above 80% [36]. The Surveyor data provides an opportunity for users to conduct their own analysis and link the data to ongoing research, as demonstrated by a 2018 paper from Okell et al. [37].

## Conclusions

The Surveyor tool provides a unique resource for the research and policy communities, allowing the user to access summary information of thousands of samples from hundreds of geographic sites across the world, whilst still maintaining the depth of detail for those users who wish to investigate further a particular location. A key resource is the full data set that supports the tool and this can be freely downloaded. The dataset can be explored for local and national prevalence of resistance markers, and the rich resource of information it contains has been highlighted here. To date, the Surveyor has had over 3000 unique viewers.

Through developing this application, the WWARN software engineering team built a flexible open-source framework of over 200,000 lines of code that can be adapted for many different types of geospatial data. This learning was subsequently employed to launch two other surveyors. In April 2015, the Artemisinin Molecular Surveyor was launched, displaying data on the artemisinin resistance markers in the *pfkelch 13* gene [38]. In 2016, the Vivax Surveyor was launched which summarizes the prevalence of chloroquine resistance in *Plasmodium vivax* [39].

One limitation of extracting data from the published literature is the inevitable delay between sample collection and publication date, making it hard to provide a really up-to-date combined picture of the evidence for resistance. Some of the benefits of developing this tool have been the clear identification of knowledge gaps, both geographically and temporally which could direct scarce resources to the regions of highest concern with respect to drug resistance surveillance and inform decisions on the use of drugs in preventive interventions.

## Additional files

**Additional file 1.** Inclusion and exclusion criteria for the literature review.

**Additional file 2.** All studies in the Surveyor Jan 2001–May 2017.

**Additional file 3.** All source publications for the data presented in Fig. 6.

## Abbreviations

ACT: artemisinin based combination therapy
*Pfcrt*: *P. falciparum* chloroquine resistance transporter
*Pfmdr1*: *P. falciparum* multidrug resistance transporter 1
*Pfpm*: *P. falciparum* plasmepsin
*Pfdhfr*: dihydrofolate reductase
*Pfdhps*: dihydropteroate synthase
CNV: copy number variation
AL: artemether–lumefantrine
ASAQ: artesunate–amodiaquine
CQ: chloroquine
MQ: mefloquine
