# Supplementary material for: An online mapping database of molecular markers of drug resistance in Plasmodium falciparum: the ACT Partner Drug Molecular Surveyor
Source: Malar J. 2019 Jan 18;18:12. doi: 10.1186/s12936-019-2645-x (PMC6339428; doi:10.1186/s12936-019-2645-x)
Supplement: Supplementary file 1 — Additional file 1. Inclusion and exclusion criteria for the literature review. [file 12936_2019_2645_MOESM1_ESM.docx]

**Inclusion criteria for publications:**

- At least one *pfcrt*, *pfmdr1* *plasmepsin2*, *pfdhfr* or *pfdhps* genotype/haplotype/copy number from isolate/infection
- Original data
- Baseline/pre-treatment infections
- Marker prevalence must be linked to study site/country

**Exclusion criteria for publications:**

- Publication not accessible
- Publication in another language than English, French, Spanish or Italian
- Review articles
- Prevalence data that have been previously published (data from original publication is included)
- Cultured strains (e.g. 3D7), genetically manipulated strains, strains that have been adapted to long-term culture in vitro (not representative of original infection)
- Non-human *P. falciparum* infections (Anopheles, monkeys)
- Post-treatment infections
- Biased selection of baseline samples (e.g. only treatment failures, half resistant and half sensitive in vitro tested isolates)
- Regionally pooled marker prevalence data (i.e. from West Africa) where the origin of the infection cannot be deduced on at least country level
- Marker prevalence (%) presented in article, without sample size
- parasites isolated from placenta
- patients with severe malaria who received treatment previously
- results previously published using another method
- not possible to distinguish which isolates originates from treated and untreated patients
- data only presented as haplotypes of *pfcrt* AND *pfmdr1* grouped together, not possible to separate marker prevalence from single genes
- case study of traveler that had self-medicated
- pooled analysis of previously published data
- prevalence includes both pre- and posttreatment isolates
